# Temporal determinants of tumour response to neoadjuvant rectal radiotherapy

**DOI:** 10.1371/journal.pone.0254018

**Published:** 2021-06-30

**Authors:** Kendrick Koo, Rachel Ward, Ryan L. Smith, Jeremy Ruben, Peter W. G. Carne, Hany Elsaleh

**Affiliations:** 1 Radiation Oncology, Alfred Health, Melbourne, Victoria, Australia; 2 Colorectal Surgery Unit, Alfred Health, Melbourne, Victoria, Australia; 3 Cabrini Monash University Department of Surgery, Melbourne, Victoria, Australia; Kalinga Institute of Medical Sciences, INDIA

## Abstract

**Introduction:**

In locally advanced rectal cancer, longer delay to surgery after neoadjuvant radiotherapy increases the likelihood of histopathological tumour response. Chronomodulated radiotherapy in rectal cancer has recently been reported as a factor increasing tumour response to neoadjuvant treatment in patients having earlier surgery, with patients receiving a larger proportion of afternoon treatments showing improved response. This paper aims to replicate this work by exploring the impact of these two temporal factors, independently and in combination, on histopathological tumour response in rectal cancer patients.

**Methods:**

A retrospective review of all patients with rectal adenocarcinoma who received long course (≥24 fractions) neoadjuvant radiotherapy with or without chemotherapy at a tertiary referral centre was conducted. Delay to surgery and radiotherapy treatment time were correlated to clinicopathologic characteristics with a particular focus on tumour regression grade. A review of the literature and meta-analysis were also conducted to ascertain the impact of time to surgery from preoperative radiotherapy on tumour regression.

**Results:**

From a cohort of 367 patients, 197 patients met the inclusion criteria. Complete pathologic response (AJCC regression grade 0) was seen in 46 (23%) patients with a further 44 patients (22%) having at most small groups of residual cells (AJCC regression grade 1). Median time to surgery was 63 days, and no statistically significant difference was seen in tumour regression between patients having early or late surgery. There was a non-significant trend towards a larger proportion of morning treatments in patients with grade 0 or 1 regression (p = 0.077). There was no difference in tumour regression when composite groups of the two temporal variables were analysed. Visualisation of data from 39 reviewed papers (describing 27379 patients) demonstrated a plateau of response to neoadjuvant radiotherapy after approximately 60 days, and a meta-analysis found improved complete pathologic response in patients having later surgery.

**Conclusions:**

There was no observed benefit of chronomodulated radiotherapy in our cohort of rectal cancer patients. Review of the literature and meta-analysis confirms the benefit of delayed surgery, with a plateau in complete response rates at approximately 60-days between completion of radiotherapy and surgery. In our cohort, time to surgery for the majority of our patients lay along this plateau and this may be a more dominant factor in determining response to neoadjuvant therapy, obscuring any effects of chronomodulation on tumour response. We would recommend surgery be performed between 8 and 11 weeks after completion of neoadjuvant radiotherapy in patients with locally advanced rectal cancer.

## Introduction

Neoadjuvant radiotherapy has been established as a key part of the treatment algorithm for locally advanced rectal adenocarcinomas and has been demonstrated to reduce the risk of local recurrence [1], with the addition of adjuvant chemotherapy further enhancing this effect [2]. A recent study from Canberra demonstrated that rectal cancer patients receiving their radiotherapy later in the day have improved pathological response to treatment, though this impact is confined to patients having earlier (<7 weeks) surgery [3]. This has been impetus to perform a replication study exploring the impact of temporal factors on radiotherapy for rectal carcinoma, with two separate factors being of particular interest–delay between radiotherapy and surgery and the timing of radiotherapy during the day.

It has long been appreciated that a longer delay to surgery after completion of neoadjuvant accelerated hypofractionated radiotherapy without concurrent chemotherapy results in greater histopathological tumour response though without survival advantage [4]. It is clear that early surgery is not ideal, but the optimal delay time has not been precisely defined, with a broad range of 6–11 weeks provided in ASTRO consensus guidelines [5]. Two large retrospective reviews looking at conventionally fractionated neoadjuvant chemoradiotherapy have suggested a gap of 8 weeks to maximise tumour response [6, 7] though other papers have suggested that delays of up to 10–11 weeks [8–10] further benefits tumour response without adversely affecting complication rates.

Separately, there has been growing recognition that circadian oscillations are critical for the regulation and synchronisation of cellular processes, with dysregulation of circadian rhythm leading to the development of cancer through myriad pathways, including impact upon DNA repair, cell cycle regulation and epigenetic modifications [11, 12]. This has been investigated in vitro, with restoration of normal circadian oscillations observed to reduce tumour cell growth in melanoma and colon adenocarcinoma cell lines [13]. Given this evidence, there have been attempts to improve the efficacy of currently available treatment modalities through chronomodulated therapy, where treatments are timed to leverage circadian differences, either improving tumour cell kill or reducing toxicity.

Whilst there have been attempts at chronomodulated chemotherapy as far back as 1994, with one trial demonstrating diminished side effects and improved survival in colorectal carcinoma [14], a meta-analysis has failed to find sufficient evidence to support these findings [15]. Interest in chronomodulated radiotherapy, by contrast, is much more recent. Multiple groups have investigated the clinical impact of radiotherapy treatment time in breast, head and neck and cervical tumours, finding statistically significant differences in treatment-induced toxicity between morning and afternoon treatments [16–21].

The evidence suggests temporal variables may alter tumour response to radiation therapy in rectal cancer. This study aims to replicate previous work done in rectal cancer to assess the impact of treatment time and delay to surgery on histopathological tumour regression.

## Materials and methods

We conducted a retrospective review of all patients with rectal adenocarcinoma who received long course neoadjuvant radiotherapy (≥24 fractions) with or without concurrent fluoropyrimidine chemotherapy at the Alfred Hospital and who subsequently had definitive surgery at either the Alfred or Cabrini Hospital. Patients were excluded if there was a delay of greater than 6 months (180 days) to surgery after completion of radiotherapy. Approval to conduct this project was granted by the Alfred Health Human Research Ethics Committee (reference 765/19) and Cabrini Research Governance (reference 03-24-02-20), with a waiver on the requirement for patient consent for this low-risk retrospective review.

Data was retrieved from the radiotherapy planning system (ARIA, Varian Medical Systems), Cabrini Monash Colorectal Database, and the Alfred Radiation Oncology research database. Demographic, staging, treatment, recurrence and survival data were collected from the Colorectal database, with staging according to the AJCC 7^th^ edition. Pre- and post- radiotherapy tumour and nodal stage as well as AJCC tumour regression grade, being the most accurate classification system for predicting local recurrence [22], were retrieved through a manual review of radiology and histopathology reports. Preoperative staging was determined by MRI, unless there were clinical contraindications, in which case staging was completed by endoscopic ultrasound and CT. The neoadjuvant rectal score (NAR) was calculated as previously described and used as an adjunct measure of outcome [23]. Treatment starting times were extracted from the planning system and split into “morning” and “afternoon” around 12:30 hours, which demarcated the middle of the work day. Where patients received 2 fractions in a single day to make up for missed treatments, each treatment was added to the total for the appropriate time period.

A review of the literature was conducted through a PubMed search in accordance with PRISMA principles, using the keywords “rectal”, “neoadjuvant” or “preoperative”, “radiotherapy” and “time” or “delay”. All studies presenting data for complete pathological response rates after neoadjuvant radiotherapy as well as time between completion of radiotherapy and surgery were included. Complete pathological response was targeted for analysis as it can be consistently interpreted between studies which use differing classification schemes to quantify response to neoadjuvant therapy. Results for each study were extracted and visualised in a summary figure. In studies which stratified patients into time groups, data for each group was plotted separately. Median delay in days was used where provided and imputed values used where time ranges are given. In addition to this visualisation, a standard meta-analysis was performed, aggregating a subset of papers which divide their patient cohort into earlier versus later surgery groups. Where patients were subdivided into three or more groups, the two groups with the shortest and the longest delay to surgery were selected.

All analysis was performed in the R statistical programming environment. Fisher’s exact test was used for comparisons for categorical variables, and Kruskal-Wallis for continuous variables. A logistic model was used to identify factors associated with tumour regression, with a focus on the temporal variables of interest. Cox proportional hazards models were used for survival analysis. The “meta” R package was used for meta-analysis, with a random-effects model selected due to the heterogeneity between studies, in particular the differing thresholds for “early” and “late” surgery [24].

## Results

### Overview of the data

Treatment data for 367 unique patients receiving radiotherapy for rectal adenocarcinoma between 2009–2019 were extracted from the planning system and cross referenced to the 420 patients having rectal surgical procedures in the same time period identified through the Cabrini Monash Colorectal Database. Matched data was available for 238 patients, 197 of whom received long course neoadjuvant treatment and subsequent definitive surgery. Pre-operative staging for the majority of patients was by MRI (174/197 patients, 88.3%) with the remainder having endoscopic ultrasound and CT. Patients were predominantly male (64%) and were of good performance status (ECOG-0–76.1%) with a median age of diagnosis of 64 years (range 26–85). Anterior resections (ultralow and low) were the most common operation, followed by abdominoperineal resections. (Table 1).

**Table 1:** Baseline clinicopathologic characteristics of 197 rectal adenocarcinoma patients.

	n (%)
**Sex**	
**F**	71 (36.0)
**M**	126 (64.0)
**ECOG**	
**0**	148 (76.1)
**1**	37 (18.8)
**2**	9 (4.6)
**3**	3 (1.5)
**TNM stage**	
^I	3 (1.52)
**II**	73 (37.1)
**III**	108 (54.8)
**IV**	13 (6.6)
**Surgical Procedure**	
^ULAR	116 (58.9)
^APR	38 (19.3)
**Low anterior resection**	17 (8.6)
**Proctocolectomy**	5 (2.5)
**Other**	21 (10.7)
**Fractionation**	
**50.4Gy/28 fractions**	179 (90.9)
**50Gy/25 fractions**	17 (8.6)
**48.6Gy/27 fractions**	1 (0.5)
**Concurrent chemotherapy**	
**Yes**	195 (99.0)
**No**	2 (1.0)

^3 patients with stage I disease had received neoadjuvant treatment at the discretion of the treating clinician. ULAR: Ultra-low anterior resection. APR: abdominoperineal resection.

Of the 197 patients, all but two received concurrent fluoropyrimidine chemotherapy as part of their neoadjuvant regimen. The most common radiotherapy prescription was 50.4Gy/28 fractions (179 patients, 90.9%) with a smaller number (17 patients, 8.6%) receiving 50Gy/25 fractions. One patient received a slightly truncated regimen of 27 fractions. Neoadjuvant treatment was completed in a median of 38 days (range 32–49 days) with only 6 patients (3.0%) taking longer than 6 weeks to complete treatment.

T downstaging was observed in more than half of all patients following neoadjuvant treatment, and significant histological tumour regression (AJCC 0 or 1) was seen in 90 patients (46%) (Table 2). As expected, histologic tumour regression was strongly correlated to T downstaging (p < 0.001) and decrease in maximal tumour dimension (p < 0.001) but was not correlated with N downstaging.

**Table 2:** Tumour downstaging in response to neoadjuvant treatment.

	Pre-neoadjuvant treatment	Post-neoadjuvant treatment
	n (%)	n (%)
T stage		
T0	0 (0)	46 (23)
T1	0 (0)	13 (7)
T2	10 (5)	47 (24)
T3	172 (87)	89 (45)
T4	15 (8)	2 (1)
Patients with T downstaging		**109 (55)**
N stage		
N0	82 (42)	143 (73)
N1	69 (35)	39 (20)
N2	41 (21)	15 (8)
N3	5 (2)	0 (0)
Patients with N downstaging		**85 (43)**
AJCC tumour regression grade		
AJCC 0		46 (23)
AJCC 1		44 (22)
AJCC 2		70 (36)
AJCC 3		37 (19)

Tumour and nodal stage pre- and post- neoadjuvant treatment, as well as AJCC regression grades, for 197 rectal adenocarcinoma patients.

### Delay to surgery

Median time to definitive surgery following neoadjuvant treatment was 63 days (range 24–164 days). No patient had positive mucosal resection margins, and circumferential margins were positive in only 4 patients (2%). There were no statistically significant differences in the complication rate between patients having “late” vs “early” surgery when stratified by the median of 63 days though there were non-significant trends towards more pelvic collections and small bowel obstruction in the late surgery group (Table 3). There were no differences in rates of wound or anastomotic complications.

**Table 3:** Complication rates comparing early and late surgery groups.

	Early surgery < = 63 days (n = 102)	Late surgery >63 days (n = 95)	p
	n (%)	n (%)	
Any surgical complication	27 (26.5)	22 (23.2)	0.623
• Prolonged ileus (>1 week)	9 (8.8)	3 (3.2)	0.137
• Urinary retention	8 (7.8)	4 (4.2)	0.376
• Pelvic collection	2 (2.0)	7 (7.4)	0.092^
• Wound complications	3 (2.9)	6 (6.3)	0.318
• Anastomotic leak	4 (3.9)	4 (4.2)	1.000
• Small bowel obstruction	1 (1.0)	6 (6.3)	0.058^
Any medical complication	12 (11.8)	9 (9.5)	0.650

Comparison of complication rates between early and late surgery groups. The most common surgical complications have been detailed separately, in order of frequency. Note that the numbers of specific complications do not add up to the total number of surgical complications as only the most common complications are listed, and some patients had more than one complication. ^indicates p < 0.1

No statistically significant between group differences were identified for age, sex, performance status or disease stage. There was a non-significant trend towards more abdominoperineal resections (27%, 26/95 vs 11%, 11/102) and fewer ultralow anterior resections (52%, 49/95 vs 65%, 66/102) in the late surgery group (p = 0.052).

**Fig 1:**
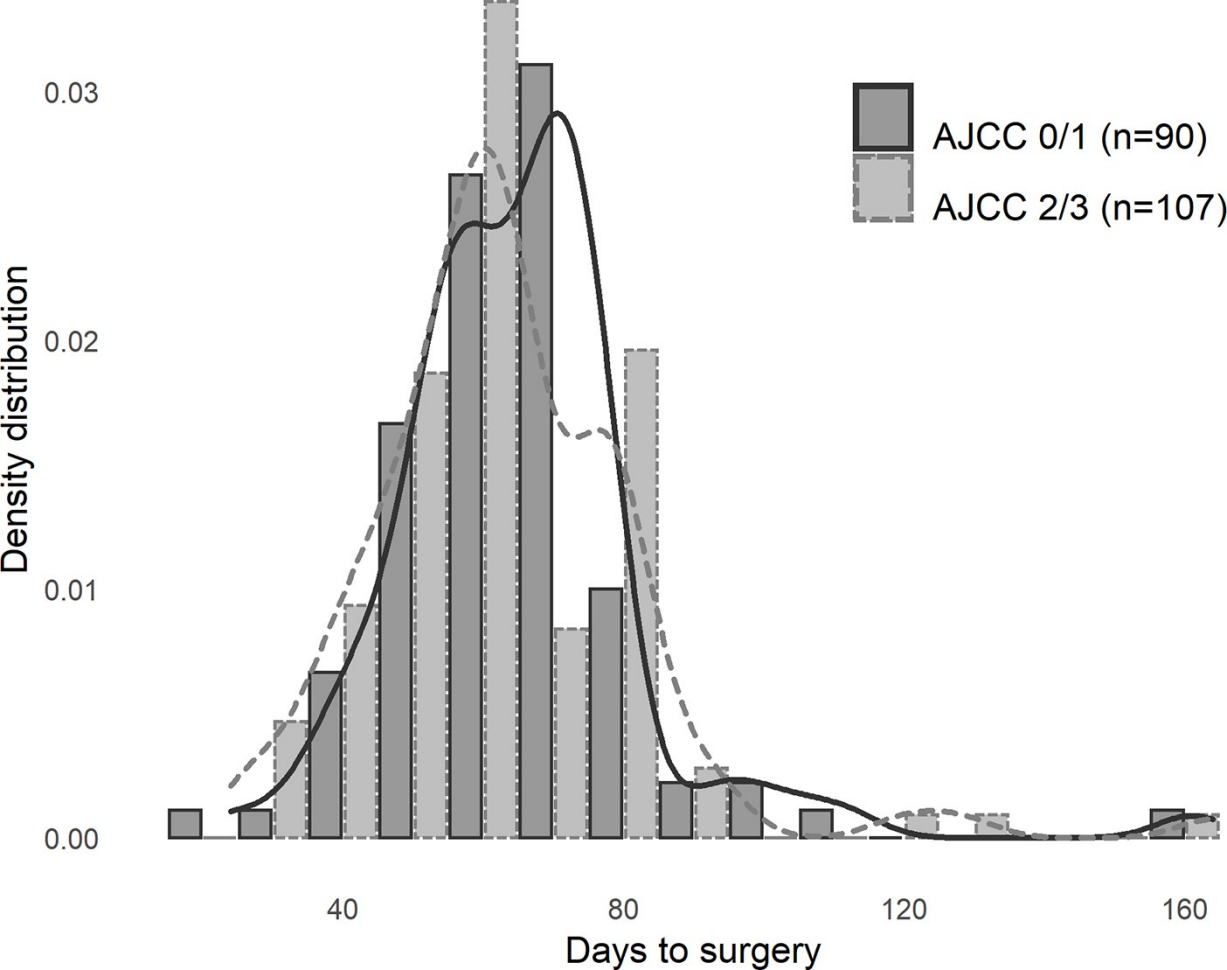
Delay to surgery by tumour regression grade. Histogram, in 5-day bins, illustrating distribution of time between completion of radiotherapy and surgery, stratified by AJCC regression grade, overlaid by a kernel density plot. No significant between group differences (p = 0.357).

There was no statistically significant correlation between time to surgery and tumour regression grade (Fig 1) and no difference in NAR score between early and delayed surgery groups (mean 15.0 vs 14.2, p = 0.706).

A range of cut-offs at 10-day intervals from 40 to 100 days for demarcation of patients into “early” and “late” surgery groups were trialled. No cut-off demonstrated statistical significance for tumour regression grade or NAR score.

### Radiotherapy treatment time

**Fig 2:**
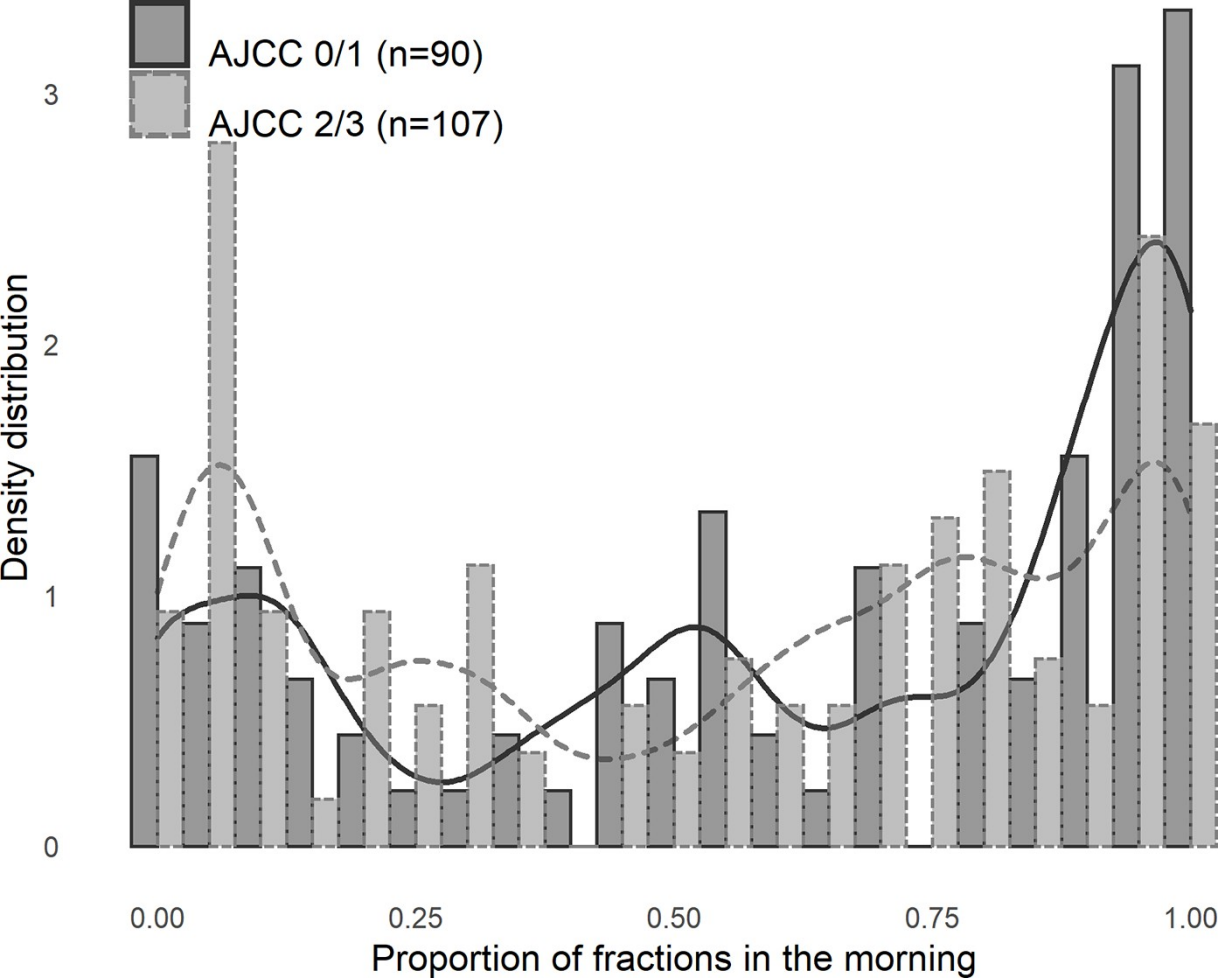
Proportion of morning radiotherapy treatments by tumour regression grade. Histogram, in bins of 0.05 (5%), illustrating distribution of proportion of radiotherapy fractions in the morning (before 1230h) per patient, stratified by AJCC regression grade, overlaid by a kernel density plot. No significant between group differences, but trend towards a bigger proportion of morning treatments for patients with AJCC 0/1 regression (p = 0.077).

A clear bimodal distribution of the proportion of morning radiotherapy treatments during each patient’s neoadjuvant course was observed (Fig 2). There was a non-significant trend towards a larger proportion of morning treatments for patients with AJCC Grade 0 or Grade 1 histologic regression when proportion of morning treatments were analysed as a continuous variable.

Patients were dichotomised into “morning” and “afternoon” treatment groups where at least 50% of treatments occurred in either the morning or afternoon. Patients in the “morning” group had poorer performance status (p = 0.015) and were older (66 years vs 59 years, p < 0.001) than those in the “afternoon” group. No statistically significant between group differences were seen for sex or disease stage. There was no difference in tumour regression grade (p = 0.385) or in NAR score (mean 14.6 vs 14.7, p = 0.925) between the dichotomised “morning” and “afternoon” groups.

To completely exclude any chronobiological effect in our data, a range of other times were used to delineate “morning” and “afternoon” treatments (i.e., 1130h, 1200h, 1300h) and tested against tumour regression grade, none of which reached statistical significance. Comparisons were also made between patients receiving at least 80% of their treatments in the morning against those having less than 20% morning treatments, and this again did not reach statistical significance.

### Combined effect of treatment time and delay to surgery

Composite groups were created to replicate the findings by Squire et al. [3], incorporating delay to surgery and time of treatments. Tumour regression grades for these composite groups were assessed (Table 4), with no statistically significant relationship found with AJCC tumour regression grade (p = 0.440).

**Table 4:** Tumour regression in composite time of treatment/time to surgery groups.

	AM treatment/early surgery (n = 55)	AM treatment/late surgery (n = 47)	PM treatment/early surgery (n = 50)	PM treatment/late surgery(n = 45)	
					p
	n (%)	n (%)	n (%)	n (%)	
AJCC 0/1	26 (43)	31 (54)	16 (39)	17 (45)	0.440
AJCC 2/3	35 (57)	26 (46)	25 (61)	21 (55)	
pCR (AJCC 0)	12 (22)	13 (26)	8 (17)	11 (24)	0.346

AJCC regression grade and complete pathological response (pCR) rates in composite time of treatment/time to surgery groups. Early and late surgery were demarcated by median time to surgery, AM/PM treatments demarcated by at least 50% of treatments occurring in either the morning or afternoon. Differences were not statistically significant.

A logistic regression model was used to explore the contributions of various clinical and treatment factors to AJCC tumour regression grade (Table 5). Advanced disease stage was found to be a negative predictor of tumour regression with no statistically significant contributions from any other variable.

**Table 5:** Logistic regression model exploring predictors for tumour regression.

	Univariate logistic regression model		
Variable	OR	CI	p
Sex	1.26	0.70–2.25	0.445
ECOG status	0.83	0.52–1.30	0.426
TNM stage	0.62	0.38–0.98	0.043*
Time to surgery	1.01	0.99–1.02	0.449
AM treatment	1.89	0.86–4.24	0.117
Treatment prolongation	0.82	0.36–1.83	0.621

AJCC grade 0/1 tumour regression (n = 90) vs AJCC grade 2/3 (n = 107). Advanced disease stage was correlated with decreased tumour regression. Positive estimates indicate an increase in grade 0/1 regression; SE = standard error

* p < 0.05.

For completeness, the impact of time of treatment and time to surgery on survival was assessed in a Cox proportional hazards model, including TNM stage, ECOG performance and tumour regression grade as covariates (Table 6). Neither temporal factor was found to be significant.

**Table 6:** Cox proportional hazards model exploring predictors for survival.

	Univariate Cox Model		
	HR	CI	p
Sex	0.77	0.35–1.73	0.532
ECOG status	1.68	1.09–2.59	0.020*
TNM stage	2.67	1.43–5.00	0.002*
Tumour regression grade	1.23	0.86–1.74	0.255
Treatment prolongation	1.21	0.42–3.50	0.725
Delay to surgery	1.01	0.99–1.03	0.284
Proportion AM treatments	2.97	0.89–9.83	0.076

HR = hazard ratio; CI = confidence interval; AHR = adjusted hazard ratio

* p < 0.05.

### Review of the literature exploring delay to surgery

**Fig 3:**
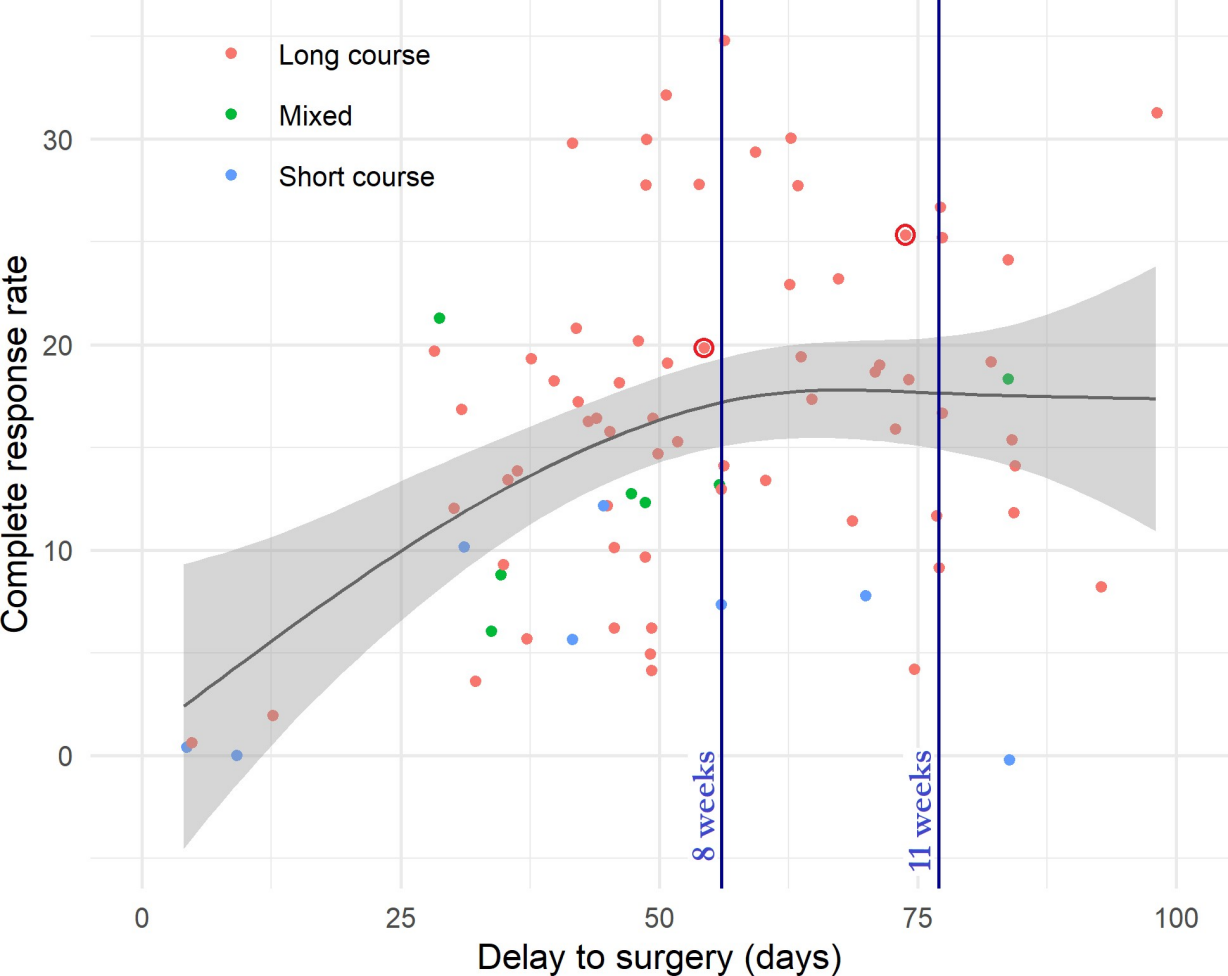
Influence of delay to surgery on complete pathological response. Delay to surgery (from completion of neoadjuvant therapy) plotted against rate of complete pathological response in 39 reviewed studies (27379 patients), coloured by included radiotherapy courses. Long course-only studies (>24 fractions) are coloured in red, short course-only studies coloured in blue, and studies including all fractionation schedules coloured in green. Data from the present study are indicated by red circles. Data points are fitted to a generalised additive model as implemented in R and this model is plotted as a trend line, with shaded areas indicating confidence intervals.

A review of the literature identified 39 papers (describing 27379 patients) which report both time delay and complete pathological response (pCR) rates to neoadjuvant rectal radiotherapy with or without the addition of chemotherapy [3, 4, 7–10, 25–58]. Three further papers were identified but excluded from visualisation as time to surgery was provided in categories rather than numerically [59–61]. The PRISMA flow chart is presented in S1 Fig. Twenty papers (51.2%) had a late surgery group with median delay to surgery greater than 70 days. Data from these papers have been summarised and is visualised in Fig 3. A trend line was computed using a generalised additive model, and this demonstrates an initial increase in pCR rate which plateaus off after a delay of approximately 60 days.

**Fig 4:**
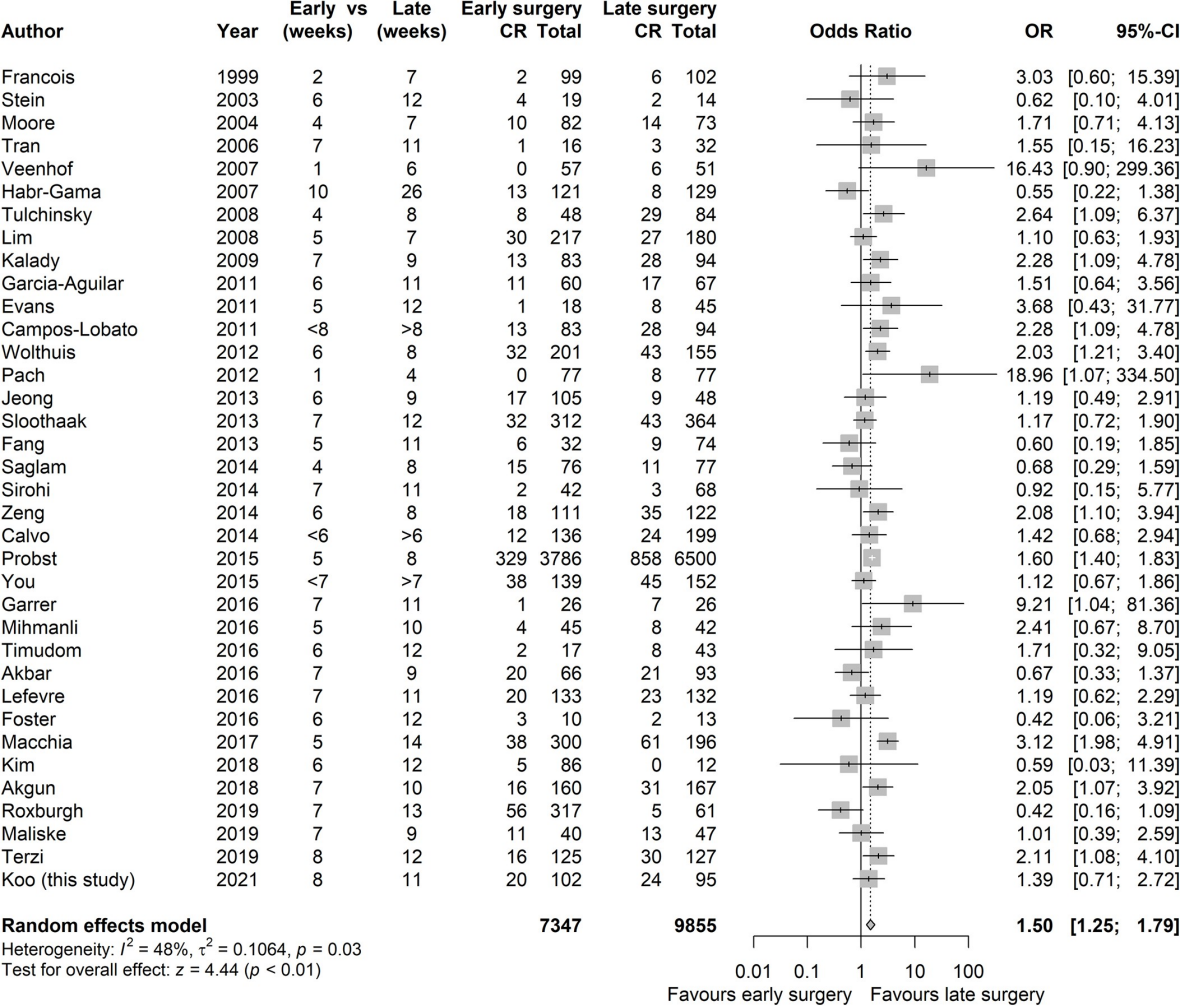
Meta-analysis of delay to surgery after neoadjuvant radiotherapy. Meta-analysis of 36 studies (17355 patients) comparing early versus late surgery after neoadjuvant radiotherapy. Studies are listed by year of publication. The median delay to surgery (in weeks) in the “early” and “late” surgery groups is provided. For the 3 studies where exact delay to surgery was unavailable, the delay threshold has been listed (with < or > signs).

A meta-analysis was performed, incorporating a total of 36 studies describing 17355 patients including the three papers not presented in Fig 3 [4, 7–10, 28–39, 41–43, 45, 47, 48, 50–52, 54–61]. There is moderate heterogeneity between studies, with an *I*^*2*^ of 48% and τ^2^ of 0.11, justifying the use of a random effects model. When the data is pooled, there is a statistically significant improvement of pCR rates in favour of later surgery, with an odds ratio of 1.50 (Fig 4).

The majority of reviewed studies which assessed surgical complications did not find any impact of surgical delay on operative complications [7, 9, 10, 28, 29, 31, 36, 39, 41–43, 51, 57, 61], with two papers finding an increase in complications with later surgery [46, 54] and another finding that later surgery decreased anastomotic and wound complications [53]. Only two of the reviewed studies assessed development of metastatic disease in the context of time to surgery after completion of radiotherapy and neither found significant between group differences [51, 53].

## Discussion

Our results do not support the use of chronomodulated radiotherapy in rectal cancer. Despite using similar methodology as Squire et al. to evaluate and test for circadian influences in tumour response, we find no chronobiological effect, with a non-significant trend towards a larger proportion of morning treatments in patients with AJCC 0/1 tumour regression, contrasting with their finding of improved tumour regression in patients with predominantly afternoon treatments [3].

Differences in clinical practice between centres might account for these differences. Squire et al. included patients having short course (25Gy/5 fractions) radiotherapy and reported a median time to surgery of 49 days, with statistically significant treatment time dependent differences in tumour regression only observed in patients having early surgery [3]. By contrast, median time to surgery in our series was 63 days—if treatment time differences in tumour regression can only be observed shortly after completion of treatment, this would not be apparent in our data.

At present, there is poor consensus in the literature as to the impact of chronomodulated radiotherapy–of the two studies addressing breast cancer, one found acute radiation side effects to be worse with afternoon treatments [17], whilst a later study found precisely the opposite, with increased dermatitis in patients receiving more morning treatments [16].This latter study identified that patients with more morning treatments were more likely to receive a boost to the tumour bed, and on multivariate analysis, there was no between group difference in acute toxicity [16]. This highlights the limits of retrospective series for chronobiological research, as there may well be systematic biases in choice of treatment time in a non-randomised study. This is also demonstrated by our finding that patients in the morning group tended to be older and of poorer performance status. Any underlying chronobiological effect is likely to be subtle and may be obscured by these biases.

In our series, time to surgery did not significantly impact tumour regression. There has been substantial controversy regarding optimal time delay between completion of neoadjuvant treatment and definitive surgery, though our meta-analysis has shown improved pCR rates with later surgery and a visualisation of the literature suggests a plateau of pCR rates after 60 days. Median time to surgery for both early and late surgery groups of patients in our cohort lie close to this plateau, and it is therefore unsurprising that no statistically significant between group difference was found. Any treatment time dependent effects which exist might be hypothesised to be most pronounced before the plateau of pCR rates, and these may therefore have been overwhelmed by the impact of time to surgery in our cohort.

Overall, this is a practice-affirming finding, as the aim at our centre is in fact to schedule surgery approximately 8 weeks after completion of neoadjuvant radiotherapy. Good pathological tumour response to neoadjuvant treatment (AJCC 0/1) is seen in a large proportion of patients, in line with previous reports [62], without any increase in surgical complication rates. There were no positive mucosal margins and an exceedingly low rate of positive circumferential margins (2%) which compares very favourably to the 17% rate in the United States National Cancer Database [63].

A few other factors of interest could not be assessed in this study. The influence of concurrent chemotherapy could not be discerned from our data as all our patients are routinely given concurrent 5-FU (or its oral equivalent capecitabine). A third temporal factor, prolongation of neoadjuvant treatment time, is also of interest as accelerated repopulation during radiotherapy treatment has been well described as a factor contributing to treatment failure [64] and has been observed after short course radiotherapy for rectal cancer [65]. We were however unable to conduct this analysis as almost all our patients had completed their radiotherapy within the appropriate timeframe.

## Conclusion

We did not identify any differences in pathological regression grade of rectal carcinomas by time to surgery or time of radiotherapy, in contrast to Squire et al. [3]. The effects of chronomodulated radiotherapy are unlikely to be large, and a randomised trial would be more appropriate in order to control for the confounding factors, especially biases which can modify time of treatment. A review of the literature finds a plateau in complete response rates after approximately 60 days, suggesting that time to surgery is a much more significant factor than time of radiotherapy, and the impact of treatment time may well have been obscured since time to surgery for the majority of patients in our study lies along this plateau. Based on our meta-analysis, we would recommend surgery between 8 and 11 weeks after completion of neoadjuvant radiotherapy for locally advanced rectal cancer.

## Supporting information

S1 Fig PRISMA flowchart. Illustration of the literature search strategy for the meta-analysis.
(DOCX)
