# Supplementary material for: Temporal determinants of tumour response to neoadjuvant rectal radiotherapy
Source: PLoS One. 2021 Jun 30;16(6):e0254018. doi: 10.1371/journal.pone.0254018 (PMC8244879; doi:10.1371/journal.pone.0254018)
Supplement: S1 Fig — Illustration of the literature search strategy for the meta-analysis. (DOCX) [file pone.0254018.s001.docx]

Records identified through database search (n = 2258).

Keywords: *“rectal”*, *“neoadjuvant”* OR *“preoperative”*, *“radiotherapy”* AND *“time”* OR *“delay”*

Records removed *before screening*:

Duplicate records removed
(n = 474)

Non-English articles (n = 238)

**Identification**

Records screened

(n = 1546)

Records excluded

(Title screen n = 1251)

(Abstract screen n = 185)

**Screening**

Full text articles assessed for eligibility

(n = 110)

Full text articles excluded

(n = 68)

Relevant studies with sufficient data reported for analysis

(n = 42)

**Included**

Studies included in meta-analysis (n = 36)

Studies included in visualisation (n = 39)

**Analysis**

**S1 Fig. PRISMA flowchart**

Illustration of the literature search strategy for the meta-analysis.
